# Scavenger Receptor CD36 Expression Contributes to Adipose Tissue Inflammation and Cell Death in Diet-Induced Obesity

**DOI:** 10.1371/journal.pone.0036785

**Published:** 2012-05-16

**Authors:** Lei Cai, Zhen Wang, Ailing Ji, Jason M. Meyer, Deneys R. van der Westhuyzen

**Affiliations:** 1 Department of Veterans Affairs Medical Center, Lexington, Kentucky, United States of America; 2 Department of Internal Medicine, Cardiovascular Research Center, Graduate Center for Nutritional Sciences, University of Kentucky, Lexington, Kentucky, United States of America; 3 Department of Molecular and Cellular Biochemistry, University of Kentucky, Lexington, Kentucky, United States of America; 4 Department of Physiology, Shandong University School of Medicine, Jinan, People’s Republic of China; Fundação Oswaldo Cruz, Brazil

## Abstract

**Objective:**

The enlarged adipose tissue in obesity is characterized by inflammation, including the recruitment and infiltration of macrophages and lymphocytes. The objective of this study was to investigate the role of the scavenger receptor CD36 in high fat diet-induced obesity and adipose tissue inflammation and cell death.

**Experimental Approach:**

Obesity and adipose tissue inflammation was compared in CD36 deficient (CD36 KO) mice and wild type (WT) mice fed a high fat diet (60% kcal fat) for 16 weeks and the inflammatory response was studied in primary adipocytes and macrophages isolated from CD36 KO and WT mice.

**Results:**

Compared to WT mice, CD36 KO mice fed a high fat diet exhibited reduced adiposity and adipose tissue inflammation, with decreased adipocyte cell death, pro-inflammatory cytokine expression and macrophage and T-cell accumulation. In primary cell culture, the absence of CD36 expression in macrophages decreased pro-inflammatory cytokine, pro-apoptotic and ER stress gene expression in response to lipopolysaccharide (LPS). Likewise, CD36 deficiency in primary adipocytes reduced pro-inflammatory cytokine and chemokine secretion in response to LPS. Primary macrophage and adipocyte co-culture experiments showed that these cell types act synergistically in their inflammatory response to LPS and that CD36 modulates such synergistic effects.

**Conclusions:**

CD36 enhances adipose tissue inflammation and cell death in diet-induced obesity through its expression in both macrophages and adipocytes.

## Introduction

Obesity, especially visceral obesity, is a well-described risk factor for the development of atherosclerotic cardiovascular disease, type II diabetes mellitus and fatty liver disease [1]. The contribution of adipose tissue (WAT) to metabolic disorders is likely linked to WAT inflammation that accompanies the recruitment and infiltration of immune cells, including adipose tissue macrophages (ATMs) and T lymphocytes [2], [3].

According to current understanding, macrophages are recruited into WAT in response to signals emanating from stressed adipocytes [4]. Nutrient excess leads to enlarged, dysfunctional adipocytes that exhibit increased free fatty acid (FFA) release as well as altered adipokine and cytokine production [5]. Alterations in lipid metabolism and the development of ER stress contribute to the activation of stress signaling pathways, such as the JNK and NFκB cascades [4], [5]. Consequently, pro-inflammatory chemokines and cytokines are released and macrophages are recruited into WAT. In obese WAT, moribund adipocytes are surrounded by macrophages in “crown-like” structures (CLS) [6], [7], [8]. Such activated macrophages release additional pro-inflammatory cytokines, such as TNFα and IL-1β, which cause further adipocyte death and stress signaling [3], [9]. Adipocyte apoptosis alone may be sufficient for macrophage recruitment [10] and is considered to be a key event contributing to dietary obesity, insulin resistance and hepatic steatosis in both humans and mouse models [10], [11]. However, the initiation steps in adipocyte stress and death remain poorly defined.

The Class B scavenger or “pattern recognition” receptor CD36, which has been extensively studied for its role in macrophage lipid accumulation and inflammatory responses [12], is a likely candidate for regulating adipose tissue apoptosis and inflammation. CD36 is a receptor for modified LDL that promotes macrophage lipid accumulation and foam cell formation through lipid uptake from modified lipoproteins [13], [14], [15]. The interaction between macrophage CD36 and oxidized LDL also triggers pro-inflammatory effects by activating NFκB signaling [16], releasing reactive oxygen species and inhibiting macrophage migration [17]. Such activation of inflammatory signaling by CD36 occurs through its interaction with Toll-like receptors (TLRs) [18], [19]. While TLR/CD36 complexes stimulate NFκB signaling and the consequent secretion of pro-inflammatory cytokines, CD36 alone can initiate downstream JNK signaling upon endocytosis of bacteria [20]. In addition to promoting phagocytic clearance of apoptotic cells by interaction with membrane-associated oxidized phosphatidylserine [21], CD36 can also promote macrophage apoptosis [22]. CD36, acting together with TLR2, triggers apoptosis in ER-stressed macrophages, usually initiated by excess lipid accumulation [22].

CD36 also functions as a fatty acid transporter, whose expression in adipocytes as well as in cardiac and skeleton muscle promotes fatty acid uptake [23], [24], [25]. CD36 is up-regulated in adipose tissue of obese subjects [26] and exerts important functions in WAT metabolism. Impaired fatty acid influx and triglyceride synthesis was reported in adipocytes lacking CD36 [27]. In response to a HFD challenge, CD36 KO mice exhibited reduced body weight gain compared to WT mice and the leaner phenotype was attributed to a reduced food intake and elevated production of leptin [28]. More recently, adipose tissue from CD36 KO mice on a HFD was reported to be more insulin sensitive, an effect associated with reduced inflammatory signaling in macrophages together with reduced macrophage migration [29], [30]. However, the possible functions of CD36 in adipocytes, including its role in adipocyte cell death and inflammation, are poorly understood. In this study, we investigated the role of CD36 in the development of obesity, focusing on its role in WAT inflammation. Our study confirms that CD36 contributes to high fat diet-induced obesity and insulin resistance and shows that CD36 enhances adipocyte cell death and WAT inflammation through its expression in both adipocytes and macrophages.

## Materials and Methods

### Animals

Mice were housed in Veterans Affairs Medical Center (VAMC, Lexington, KY) with a 12-hour light/dark cycle and all animal protocols received appropriate institutional approval (Animal Welfare Assurance Number of the Veterans Affairs Medical Center A3506-01; VMU IACUC protocols 2009-0005V, 2009-0006V). CD36 KO mice, backcrossed for 10 generations into a C57BL/6 background, were obtained from Dr. Kathryn Moore (New York University). Male CD36 KO and WT (C57BL/6) mice (9–10 weeks of age) were fed a HFD (60% kcal from fat; # D12492, Research Diets, New Brunswick, NJ, USA) for 16 weeks. Animals were housed in a pathogen-free facility and given free access to food and water. Metabolic analyses were performed in these mice monthly. Plasma lipids were determined by enzymatic kits (Wako Chemicals, Richmond, VA, USA). Cytokines and insulin were determined by ELISA sets (Invitrogen, Carlsbad, CA, USA). Glucose tolerance test (GTT): mice were fasted for 6 hours after which a bolus of D-glucose (20% solution, 1 g/kg body weight) was administrated intraperitoneally (i.p.). Blood was collected at different time points (0, 15, 30, 60, 90 and 120 min) post-glucose injection. Insulin tolerance test (ITT): mice were fasted for 6 hours after which a bolus of insulin (1 IU/kg body weight) was administered i.p. and blood was collected at various time points after insulin injection. Blood glucose levels were determined by using a Contour glucose monitoring system (Bayer HealthCare, Mishawaka, IN, USA). The fat and lean mass of WT and CD36 KO mice were determined by MRI using an Echo MRI-5000 instrument (Echo Medical System, Houston, TX, USA). The food intake was calculated by measuring food consumption for 3 consecutive days using metabolic cages (LabMaster Metabolism Research Platform, TSE Systems InC, Chesterfield, MO, USA).

### Primary Macrophage Culture

Mouse peritoneal macrophage isolation was performed as previously described [31]. Briefly, peritoneal macrophages were harvested from chow-fed, age matched WT and CD36 KO mice by lavaging the peritoneal cavity with PBS, 5 days after Bio-gel elicitation by i.p. injection with polyacrylamide gel P-100 (Bio-Rad, Hercules, CA, USA; 2% w/v in endotoxin-free water, 1 ml/mouse). Macrophages were then plated on 12-well plates in RPMI medium supplemented with 10% FBS, 50 IU/ml penicillin, 50 µg/ml streptomycin and 2 mM L-glutamine. Four hours after plating, cells were washed 3 times with PBS to remove the non-adherent cells and polyacrylamide beads. The attached macrophages were differentiated in RPMI medium supplemented with 15% L-cell conditioned medium, 10% FBS, 50 IU/ml penicillin, 50 µg/ml streptomycin and 2 mM L-glutamine for 24 hours.

### Primary Adipocyte Culture

Pre-adipocytes were isolated from the adipose stromal vascular fraction (SVF) according to a published procedure [32]. Briefly, freshly collected epididymal WAT from chow-fed animals was digested in Krebs buffer containing 1 mg/ml type I collagenase (Worthington Biochemical Corporation, Lakewood, NJ, USA) and 1% fatty acid-free BSA (Sigma, St. Louis, MO, USA) at 37°C for 1 hour. The suspension was filtered through a sterile 100-µm nylon mesh and centrifuged at 500×g for 5 min. The pre-adipocyte containing pellet fraction was washed 3 times with Krebs buffer and further with red blood cell lysis buffer to remove red blood cells. Cells were then counted and plated (1×10^5^ cells/well) in DMEM/F12 (1∶1) medium supplemented with 10% FBS, 33 µM biotin, 17 µM pantothenic acid, 50 IU/ml penicillin, 50 µg/ml streptomycin (P/S) and 2 mM L-glutamine. After confluency was reached, pre-adipocytes were differentiated for 2 days in differentiation medium containing 0.02 µM insulin, 25 nM dexamethasone, 0.5 mM IBMX (3-Isobuyl-1-methylxanthine), 2 µM Rosiglitazone, 10 µg/ml transferrin and 0.2 nM thyroid hormone T3 (Sigma, St. Louis, MO, USA).

Mature adipocytes were identified as Oil Red-O (ORO) positive cells containing 3 or more distinct lipid droplets. Adipocyte cultures showed negligible contamination by monocytes/macrophages as evidenced by negligible TNFα gene and protein expression in response to LPS (see results). The expression of CD36 in differentiated adipocytes promoted fatty acid uptake and triglyceride synthesis (Fig. S1), as shown in earlier studies [14]. The adipocytes from CD36 KO mice exhibited an unaltered gene expression of other potential fatty acid transporters, namely SR-BI, FATP4 and FABP4 (Fig. S2), suggesting a lack of compensation by any of these proteins for the CD36 deficiency in these cells.

### Primary Adipocyte and Macrophage Co-cultures

Co-cultures were performed as described previously [33].

#### 1. Contact co-culture system

Primary pre-adipocytes were seeded (1×10^5^ cells/well) in 12-well plates and differentiated for 2 days in differentiation medium as described above. Peritoneal macrophages were isolated and plated on top of the adipocyte layer. Cells were washed to remove any unattached cells after 3 hours of co-culture. The co-cultures were incubated for a further 16 hours in DMEM/F12 medium supplemented with 10% FBS, followed by treatment with LPS (10 ng/mL) for 4 hours in DMEM/F12 medium. As controls, macrophages and adipocytes cells were cultured in separate wells.

#### 2. Transwell co-culture system

Primary macrophages and pre-adipocytes were isolated and grown separately in 12-well transwell plates with a 0.4-µm porous membrane (Corning Scientific, Lowell, MA, USA). Primary pre-adipocyte were seeded on the bottom chamber (1×10^5^ cells/well) and allowed to differentiate for 2 days in 12-well plates. Primary macrophages (1×10^5^ cells/well) were then plated and grown in the top inserts in RPMI medium supplemented with 10% FBS. Following treatment of LPS (10 ng/mL) for 4 hours, medium and cells from the top insert and the bottom chamber were harvested and analyzed.

### Adipose Tissue Histology

Adipose tissue (epididymal fat pad) was fixed with 10% formalin for 24 hours at room temperature and embedded in paraffin. The tissue blocks were sectioned (5 µM), deparaffinized, and heated in Target Retrieval Solution (DAKO, Denmark) for antigen retrieval. Immuno-histochemical staining for macrophage was carried out with monoclonal rat anti-F4/80 (Serotec, Raleigh, NC, USA), followed by incubation with avidin-biotin complex (ABC kit, Vector Lab, Burlingame, CA, USA) and counterstaining with hematoxylin. Immunofluorescence staining of CD3 and perilipin were performed using monoclonal rabbit anti-perilipin (Cell Signaling, Danvers, MA, USA) or rabbit anti-CD3 (Calbiochem, San Diego, CA, USA) and Alexa-568-conjugated anti-rabbit IgG (Invitrogen, Carlsbad, CA, USA). Immunofluorescence staining of F4/80 was performed using monoclonal rat anti-F4/80 and Alexa-488-conjugated anti-rat IgG (Invitrogen, Carlsbad, CA, USA). For negative control, nonimmune IgG was used as primary antibody. Nuclei were stained with DAPI. Sections were visualized on an Olympus BX51 microscope and images were captured using equal exposures. For quantitative analysis, cells from five random fields, each containing more than 100 cells/field, were examined and counted.

### Quantitative PCR (Q-PCR)

Total RNA was isolated from cells and tissues using the standard TRIzol method (Invitrogen, Carlsbad, CA, USA). RNA was further purified with DNase I (Roche, Indianapolis, IN, USA) and RNeasy Mini Kit (QIAGEN, Valencia, CA, USA). 2 µg of RNA was reverse transcribed into cDNA using a reverse-transcription system (Promega, Madison, WI, USA). Q-PCR amplification was carried out for 40 cycles using a Power SYBR Green PCR Master Mix Kit (Applied Biosystems, Carlsbad, CA, USA) and a DNA Engine Optical 2 System (MJ Research Inc., Ramsey, MN, USA). Both an internal control (36B4/GAPDH) and negative control (minus reverse transcriptase) were included. Values of each RNA sample were normalized to 36B4 (acidic ribosomal phosphoprotein P0) or GAPDH mRNA levels.

### Statistics

Statistical significance in experiments comparing only 2 groups was determined by 2-tailed Student’s *t* test. The significance of the difference in mean values between more than 2 groups was evaluated by one-way ANOVA, followed by post hoc analysis using Tukey’s test. All significant differences (p<0.05) are given in the figures and/or figure legends. All statistical analyses were carried out using Graph Pad Prism 4 (GraphPad Software, CA, USA). Values are expressed as mean ± SD.

## Results

### CD36 KO Mice are Protected Against Diet-induced Obesity and Adipose Tissue Inflammation

**Figure 1:**
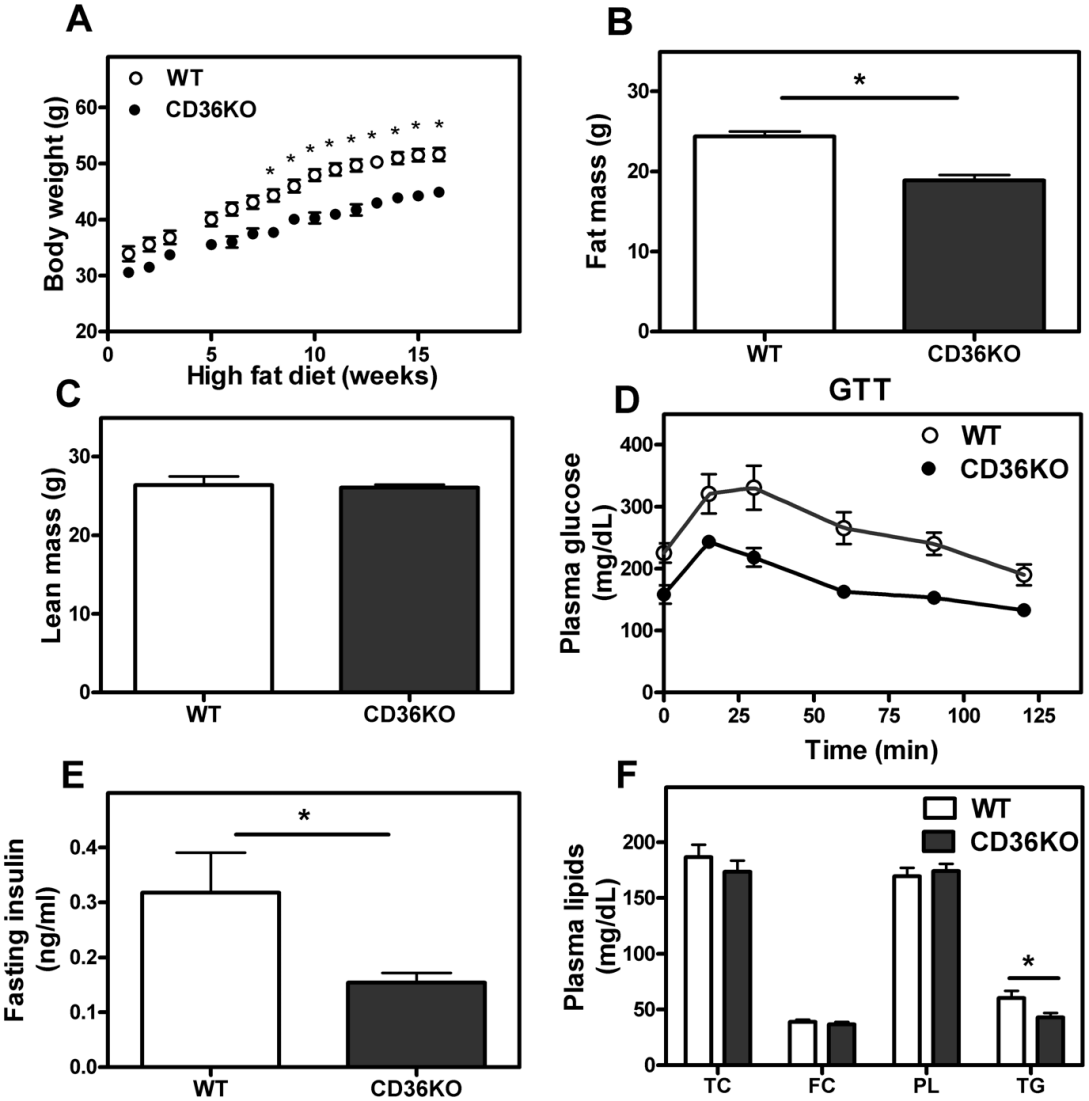
CD36 KO mice are resistant to high fat diet-induced obesity compared to WT mice. **A–C**. WT and CD36 KO male mice were maintained on a HFD for 16 wks. Body weight was measured weekly. After 16 wks on HFD, lean body mass and fat body mass were determined by MRI. Plasma was collected after 6 h of fasting. **D**. Glucose tolerance test (GTT). Mice were given a bolus of D-glucose (2 g/kg body weight) i.p. after 6 h of fasting. **E**, **F**. Fasting (6 h) insulin levels were determined by ELISA kits (BD Bioscience) and plasma lipid levels were determined by commercially available kits (WAKO). Values shown are mean ± SD (n = 7 except GTT, n = 5). *, p<0.05, CD36 KO vs. WT.

CD36 KO mice were found to be leaner than control (WT) mice following 16 weeks of a high-fat ‘obesity’ diet containing 60% (kcal) fat (Fig. 1A). MRI analysis indicated the reduced body weight of CD36 KO mice was the result of lower fat mass whereas lean body mass did not differ between the two genotypes (Fig. 1B, 1C). CD36 KO mice showed improved metabolic status as indicated by lower fasting plasma glucose, insulin and plasma triglyceride levels as well as greater glucose tolerance in response to a bolus of glucose injection (Fig. 1D–F). These findings are in line with the results of recent reports [28], [29], [30], which showed that CD36 KO mice on a high fat/high cholesterol “western” diet are also leaner with improved insulin signaling. Lipids in liver were analyzed and no significant differences in TG, PL, unesterified cholesterol, total cholesterol or FFA were observed between WT and CD36 KO mice (data not shown). To obtain further information on the metabolic status of these mice, mice were placed in metabolic cages for 1 week after 15 weeks of high fat feeding. WT and CD36 KO mice showed similar food intake (Fig. S3). CD36 KO mice showed greater insulin tolerance (Fig. S4). In comparison to mice fed a HFD, 24 week-old WT and CD36 KO mice on a chow diet did not exhibit differences in fat mass, lean mass or plasma lipid levels (Fig. S5A–C). Interestingly, CD36 KO mice displayed a slight but significantly greater glucose tolerance than WT mice on a chow diet (p<0.05, area under the curve; Fig. S5D).

**Figure 2:**
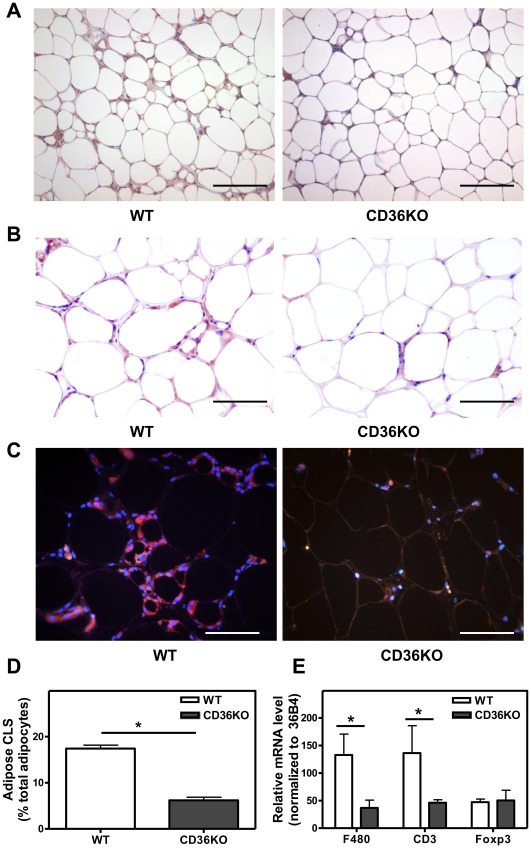
Reduced macrophage and T cell infiltration in adipose tissue from CD36 KO mice fed a HFD compared to WT mice. **A–C**. Representative images of F4/80 and CD3 stained epididymal fat sections from male WT and CD36 KO mice after 16 wks on a HFD. **A**, **B** F4/80-positive macrophages (brown). **C.** CD3-positive cells (red). **D**. The number of CLS was calculated as the percentage of adipocytes that are found in crown-like structures. For quantitative analysis, cells from 5 random fields, each containing more than 100 cells/field, were examined and counted. Values shown are mean ± SD (n = 3 mice), *, p<0.05. **E.** Adipose tissue gene expression. RNA was extracted and gene expression was determined by Q-PCR. Data was normalized to 36B4 expression. Values shown are mean ± SD (n = 5). *, p<0.05. Scale bar, 200 µm (**A**), 100 µm (**B**, **C**).

To determine whether the protection against HFD-induced obesity paralleled a reduction in AT inflammation, we evaluated the inflammatory status of adipose tissue in high-fat fed WT and CD36 KO mice. In agreement with the recent studies [29], [30], we found that epididymal fat from WT mice on a HFD showed significant F4/80-positive macrophage accumulation and increased numbers of crown-like structures which are associated with necrotic adipocytes (Fig. 2A, 2B, 2D). The presence of F4/80 positive macrophages was minimal in adipose tissue from either CD36KO or control mice fed a chow diet (Fig. S6A). In contrast, adipose tissue from CD36 KO mice demonstrated a much reduced macrophage presence. The infiltration of T lymphocytes, which are considered to contribute to macrophage recruitment [34], [35], was also assessed. T lymphocytes, immuno-stained for CD3, were found to be markedly fewer in CD36 KO tissue (Fig. 2C). CD36 KO adipose tissue showed reduced gene expression of the macrophage marker F4/80 (3-fold) and the T lymphocyte marker CD3 (2.5 fold) compared to WT tissue (Fig. 2E). Foxp3-positive regulatory T cells are highly enriched in the abdominal fat of normal mice, exerting anti-inflammatory effects, but are markedly reduced in number in diet-induced obesity [35]. Foxp3 gene expression was similar in the two genotypes indicating that the proportion of T regulatory cells in the T cell population was elevated in CD36 KO adipose tissue compared to WT controls.

**Figure 3:**
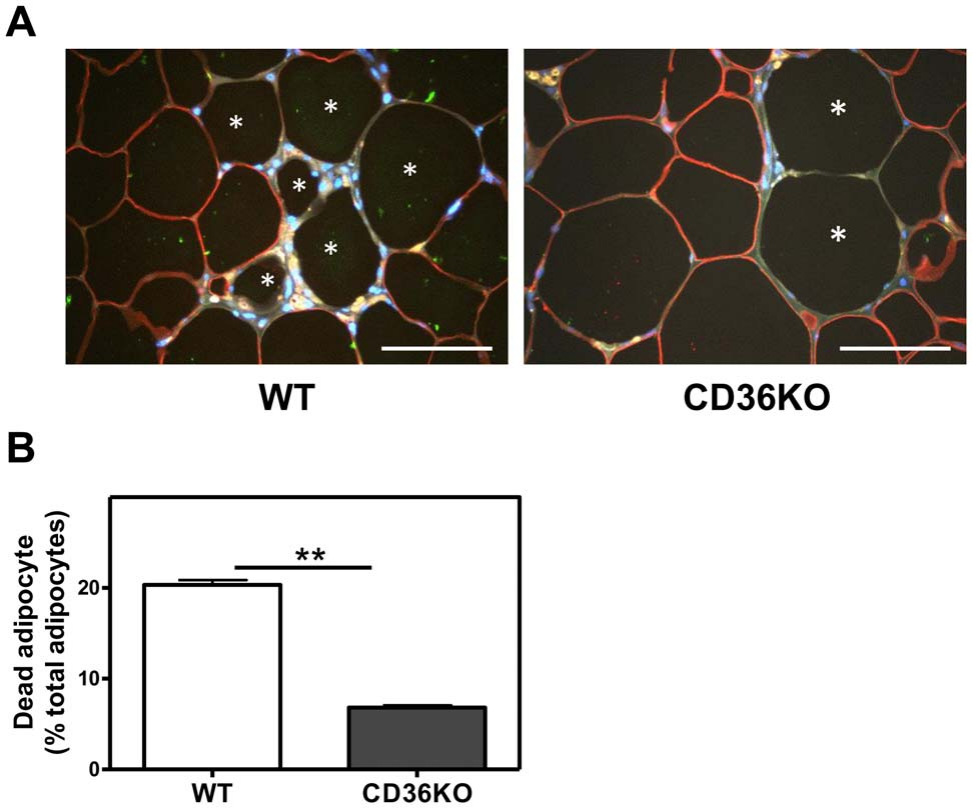
Cell death in CD36 KO adipose tissue is reduced compared to WT mice. A . Representative images of F4/80 and perilipin stained epididymal fat sections from male WT and CD36 KO mice after 16 wks on a HFD. Viable adipocytes are shown as perilipin-positive (red) and macrophages as F4/80-positive (brown). Cell nuclei were stained with DAPI (blue). Stars (*) indicate the cells devoid of perilipin expression. **B**. Quantification of dead adipocytes (perilipin-negative cells) expressed as a percentage of total adipocytes. For quantitative analysis, cells from 5 random fields, each containing more than 100 cells/field, were examined and counted. Values shown are mean ± SD (n = 3 mice), *, p<0.05. Scale bar, 100 µm.

Crown-like structures, characterized by macrophages surrounding a moribund adipocyte, are frequently associated with obesity. Co-staining with the macrophage marker F4/80 and the adipocyte specific marker perilipin [6] showed that WAT from WT mice contained substantial numbers of perilipin-negative cells, which lack the intact perilipin layer that surrounds the normal adipocyte lipid droplet [6] (Fig. 3A). Perilipin-negative cells were predominantly localized in CLS, surrounded by F4/80-positive macrophages. Compared to WT WAT, CD36-deficient adipose tissue showed reduced numbers of perilipin-negative cells, indicative of reduced adipocyte cell death (Fig. 3A, 3B)**.** Given that adipocyte cell death is a contributor to inflammation and dietary obesity, elucidation of the molecular mechanisms responsible for CD36-dependent adipocyte cell death is key to understand how CD36 influences diet induced obesity.

**Figure 4:**
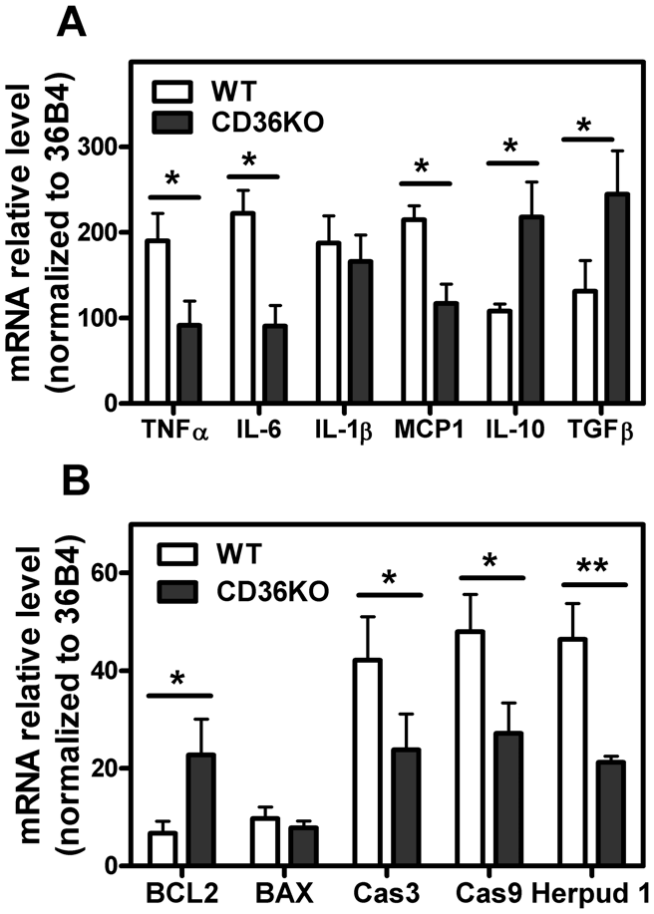
CD36 induces pro-inflammatory and pro-apoptotic gene expression in adipose tissue. Epididymal fat from WT and CD36 KO mice after 16 wks on a HFD. RNA was isolated and Q-PCR was performed. **A**. Expression of genes associated with inflammation. **B**. Genes associated with cell death. Data was normalized to 36B4 expression. Values shown are mean ± SD (n = 5), *, p<0.05; **, p<0.001.

Reduced immune cell infiltration in CD36 KO WAT was accompanied by a reduced inflammatory response to diet-induced obesity compared to WT mice. CD36 KO mice had significantly reduced pro-inflammatory cytokine gene expression in WAT compared to WT mice, shown by lower IL-6, TNFα and MCP-1 expression (Fig. 4A). In addition, we showed a substantially greater expression of the anti-inflammatory cytokines IL-10 and TGFβ in CD36 KO adipose tissue (Fig. 4A). We next examined the relationship between WAT inflammation and cell death. WAT from mice lacking CD36 had markedly lower expression of the apoptosis-related genes measured, such as caspase 3 and caspase 9 (Fig. 4B). The expression of BAX and BCL-2, two proteins that play important roles in the initiation of the mitochondrial pathway of apoptosis [11], [36], was also examined. BCL-2 gene expression was greater in CD36 KO AT, with a corresponding lower BAX/BCL-2 gene expression ratio, indicative of reduced sensitivity to FAS-mediated apoptosis [11], [37] in the CD36 KO tissue. Consistent with the reduced level of inflammation in AT lacking CD36, the expression of the ER stress gene Herpud1 was also lower. The attenuated pro-inflammatory response gene expression observed in CD36 KO mice only occurred in high fat feeding conditions and not in mice fed a chow diet (Fig. S6B).

### CD36 Expression Modulates the Adipocyte Inflammatory Response

**Figure 5:**
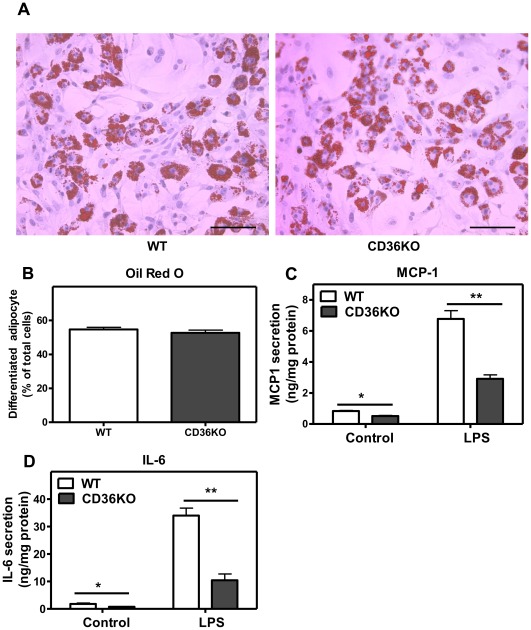
CD36 promotes adipocyte inflammatory cytokine and chemokine production in response to LPS. Adipocytes were differentiated from the SVF in culture wells as described in Materials and Methods. **A**. Lipid accumulation in adipocytes was determined by Oil Red O staining. **B**. Differentiated (Oil Red O-positive) cells were calculated as a percentage of total cells. **C**, **D**. Cytokine levels in culture medium was determined following 4 h treatment of cells with LPS (10 ng/mL). Values shown are mean ± SD (n = 4) from a representative experiment, *, p<0.05; **, p<0.001. Scale bar, 100 µm.

Consistent with recent studies [22], [38], we found that CD36 expressed in mouse peritoneal macrophages promotes the expression of pro-inflammatory cytokines (TNFα, IL-6, IL-1β, MCP-1), TNF receptor associated factor (TRAF), as well as genes associated with apoptosis (Caspase 3 & 9, FAS) and ER stress (CHOP, Calnexin), in response to LPS treatment (Fig. S7). In addition to macrophages, adipocytes also contribute to the increased expression of inflammatory mediators in WAT of obese mice. However, the possible role of adipocyte CD36 in regulating adipocyte inflammatory responses is not known. We therefore measured inflammatory responses to LPS in primary adipocytes differentiated from SVF of WAT obtained from WT and CD36 KO mice. A similar proportion (approximately 55%) of SVF cells from WT and CD36 KO mice differentiated into highly lipid-loaded adipocytes as shown by ORO staining (Fig. 5A, 5B). Untreated primary adipocytes secreted low levels of IL-6 and MCP-1; whereas LPS-treated cells showed markedly induced MCP-1 and IL-6 expression (Fig. 5C, 5D). Under both basal conditions and in response to LPS treatment, the CD36 KO cells exhibited a significantly reduced secretion of IL-6 and MCP-1 compared to WT cells (Fig. 5C, 5D). Together, these data extend the current understanding of the function of CD36 in WAT, indicating that, as in the case of macrophages, the inflammatory response in adipocytes was markedly attenuated in the absence of CD36.

### Adipocytes and Macrophages Interact Synergistically to Affect the Inflammatory Response

**Figure 6:**
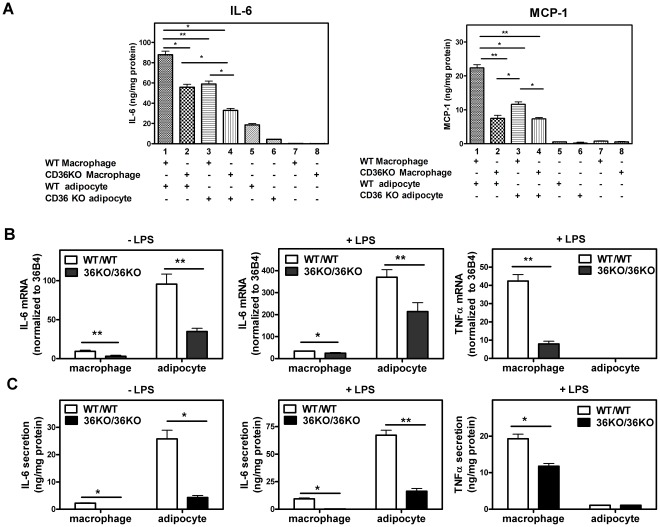
Macrophages and adipocytes display a synergistic and CD36- dependent cytokine response to LPS. A . Contact co-culture. Adipocytes were differentiated from the SVF of WT and CD36 KO mice as described in Materials and Methods. Peritoneal macrophages isolated from WT and CD36 KO mice were then layered and cultured on top of the differentiated adipocytes and co-cultured for 16 h. LPS (10 ng/mL) was then applied to the co-cultures for 4 h after which medium and cells were collected for cytokine determination. **B**, **C**. Non-contact co-culture in transwells. Primary pre-adipocytes were seeded in the bottom chamber and differentiated into mature adipocytes. Primary peritoneal macrophages were then seeded in the transwell inserts and cells were then co-cultured for 16 h. For LPS treatment groups, both adipocytes and macrophages were exposed to LPS (10 ng/mL) for 4 h after which cellular gene expression was measured by Q-PCR (B). Medium was collected and secreted cytokines were measured by ELISA (C). Values shown are mean ± SD (n = 4). *, p<0.05, **, p<0.001.

Recent studies using co-culture systems *in vitro* indicate that interactions between adipocytes and macrophages play an important role in the inflammatory response in WAT [39]. To determine if CD36 influences the inflammatory response in macrophage/adipocyte co-culture systems, we first investigated “contact” co-cultures in which peritoneal macrophages are layered and cultured on primary differentiated adipocytes. Following LPS treatment, macrophages or adipocytes cultured separately secreted relatively low amounts of IL-6 and MCP-1 into the medium (Fig. 6A, lanes 5–8), compared to markedly increased levels of secretion in co-cultures containing both cell types (Fig. 6A, lanes 1–4). Interestingly, the highest levels of IL-6 and MCP-1 were observed when CD36-positive macrophages and adipocytes were co-cultured (lane 1). In the case of IL-6, the lowest levels were observed in co-cultures of CD36 KO macrophages and CD36 KO adipocytes (lane 4), while intermediate levels were found in “mixed” cultures where one of the cell types was CD36 KO (lanes 2, 3). In the case of MCP-1, the absence of CD36 in one or both cell types markedly reduced secretion. To assess if the observed differences in gene expression between the different co-cultures could be explained simply by varying ratios of adipocytes to peritoneal macrophages in the co-cultures, gene expression of F4/80 and adiponectin, which are expressed specifically in macrophages and adipocytes, respectively, was determined. As expected, adipocytes cultured alone showed greater levels of adiponectin gene expression than co-cultures of adipocytes and macrophages (Fig. S8A). However, similar levels of adiponectin expression were found in the co-cultures that differed in their adipocyte CD36 genotype. Similarly, greater F4/80 expression was observed in macrophages cultured alone compared to co-cultures, while similar levels of F4/80 expression were observed in co-cultures that differed in their macrophage CD36 genotype (Fig. S8B). These results provide evidence that the different co-cultures have similar adipocyte to macrophage cell ratios (Fig. S8A, 8B). Overall, these results indicate that CD36 serves as a key regulator of macrophage/adipocyte interactions that determine the inflammatory status in adipose tissue.

To assess the distinct contribution(s) of macrophages and adipocytes to the enhanced cytokine expression observed in the co-cultures, we used “non-contact” co-cultures in transwell dishes. In these experiments we compared co-cultures of CD36 KO adipocytes and CD36 KO macrophages with co-cultures of WT adipocytes and WT peritoneal macrophages. As expected, CD36-deficiency was accompanied by reduced adipocyte IL-6 gene expression (Fig. 6B) and protein levels (Fig. 6C) in the adipocyte compartment, both before and following LPS treatment. Noticeably, adipocytes showed markedly greater expression of IL-6, both at the transcriptional and protein level, compared to macrophages either before or after LPS stimulation. It is likely that IL-6 can diffuse between the two transwell compartments. However, the much greater levels of IL-6 in the adipocyte compartment indicate its production is predominantly from adipocytes. This conclusion is in line with the greater IL-6 gene expression found in adipocytes compared to macrophages. In contrast to IL-6, gene expression and protein secretion of TNFα was substantially higher in macrophages than in adipocytes. Together, these data support a major role for adipocytes in CD36-dependent inflammatory cytokine production during adipose tissue inflammation.

## Discussion

This study investigated the role of CD36 expressed in adipose tissue. We showed that CD36 enhances both adipose tissue inflammation and adipocyte cell death in mice in response to high fat feeding. CD36 expression in cultured primary adipocytes was shown to enhance adipocyte inflammatory cytokine expression, indicating that CD36 contributes to inflammatory responses in adipocytes as well as in macrophages. In addition, inflammatory responses in these two cell types are regulated in a synergistic and CD36-dependent manner.

Our studies confirmed earlier reports showing that mice lacking CD36 were significantly protected against HFD-induced obesity associated with increased plasma insulin and glucose levels and reduced glucose tolerance [28], [29], [30]. In one study, the CD36 KO phenotype was attributed to elevated leptin secretion and subsequent decreased food intake [28]. However, we found no difference in food intake between WT and CD36 KO mice during high fat feeding, in agreement with a more recent study [30]. The explanation for why CD36 KO mice show reduced obesity despite having a similar food intake to WT mice is not known. One possibility is that CD36, a fatty acid transporter, may contribute to fatty acid absorption in the small intestine [25]. However, CD36 KO mice showed unaltered intestinal fatty acid uptake, except for the very long chain fatty acids [40], [41]. A decreased chylomicron secretion with a subsequent delay in fat absorption was observed in CD36 KO mice [42]. While such a delay in CD36 KO mice may not affect the overall lipid absorption in chow-fed mice, CD36 may become an important contributor to fatty acid absorption under high fat feeding conditions, a possibility that warrants further study.

Increased adipocyte cell death is commonly observed in obese humans and mice [11]. Recent studies indicate that adipocyte apoptosis is a key initiating event for macrophage infiltration into adipose tissue and for insulin resistance [11]. Inactivation of the key apoptosis-regulating molecules, Bid or FAS (CD95), resulted in reduced macrophage infiltration and improved systemic insulin sensitivity in mice [11], [43]. Importantly, adipocyte apoptosis alone appeared to be sufficient to induce macrophage infiltration in mouse adipose tissue [10]. Our findings indicate that CD36 promotes adipocyte and macrophage cell death in adipose tissue during diet-induced obesity.

Interestingly, the increased adipose cell death observed in WT mice on a HFD was evidently not accompanied by decreased cell number. On the contrary, these mice are more obese than their CD36 KO counterparts, despite having adipocytes that do not differ in size from the adipocytes in CD36 KO mice. This may be explained by a continuous remodeling of adipose tissue that involves the elimination and then replacement of adipocytes from progenitor cells [10], [44]. Thus, the loss of the adipocytes may be counterbalanced by newly differentiated adipocytes. Interestingly, a high degree of adipocyte death (approximately 80%) was reported in mouse epididymal adipose tissue after 16 weeks of high-fat feeding, while at 20 weeks of feeding only 16% of adipocytes were necrotic [7]. These findings support the concept that progression of obesity is associated with an early phase involving adipocyte hypertrophy with subsequent apoptosis and necrosis, and a later stage involving hyperplasia and tissue remodeling [7]. Our findings suggest that CD36 is likely to affect adipose tissue remodeling and expansion by promoting adipocyte cell death. As expected, reduced cell death in adipose tissue in CD36 KO mice was accompanied by decreased inflammation and a subsequent reduction in macrophage and T cell infiltration. The reduced cell death and inflammatory responses, as well as reduced obesity in CD36 KO mice were accompanied by improved insulin sensitivity, as recently reported by others [29], [30].

The contribution of adipocyte CD36 to the inflammatory response has not been previously described. We report that CD36 expression in primary adipocytes markedly exacerbated the pro-inflammatory cytokine response to LPS. In these experiments, adipocytes produced very significant amounts of cytokines in a CD36-dependent manner. Interestingly, adipocytes and macrophages produce distinct patterns of cytokines in response to LPS challenge. IL-6 was predominantly produced by adipocytes while TNFα was mainly produced by macrophages. Importantly, CD36 regulated cytokine production from both cell types, providing strong evidence that CD36 functions as an important regulator of adipose tissue inflammation through its effects in both adipocytes and macrophages.

Adipocyte/macrophage co-culture experiments have shown that these two cell types can act in a synergistic manner to promote a pro-inflammatory response [33], [39]. Our finding that CD36 expression in both cell types markedly impacted such a synergistic inflammatory response therefore provides new insight into the etiology of obesity in which interactions between adipocytes and macrophages are important determinants. The signals responsible for triggering inflammatory response in adipocytes during obesity, as well as the mechanism by which CD36 might promote this, are not yet understood. One possibility is that excessive lipid accumulation results in ER stress and a subsequent unfolded protein response (UPR) that activates inflammatory signaling [45]. CD36 may promote such a response by enhancing fatty acid and modified lipoprotein uptake into cells and by increasing triglyceride synthesis in cells (Fig. S1) [12]. The interaction of oxidized LDL with CD36 is known to stimulate inflammatory signaling in macrophages and as a consequence suppresses insulin signaling by attenuating AKT and IRS-1 activation [29], [30]. Alternatively, CD36-mediated fatty acid uptake may serve to regulate adipocyte function through activation of PPARγ, a nuclear receptor responsible for adipocyte differentiation and adipogenesis [46], [47].

In summary, the current study provides new understanding of the role of CD36 in adipose tissue function and diet induced obesity. First, CD36 expression influences inflammatory responses in adipocytes as well as in macrophages by promoting pro-inflammatory cytokine expression. Second, adipocytes and macrophages contribute to the regulation of adipose tissue inflammation in a synergistic and CD36-dependent manner. We conclude that CD36 expression in both macrophages and adipocytes plays an important contributory role in diet-induced adipose tissue inflammation and adipocyte cell death**.**

## Supporting Information

Figure S1 **Reduced FFA uptake and triglyceride synthesis in CD36-deficient adipocytes compared to WT control.** **A.** FFA uptake. Mature primary WT and CD36 KO adipocytes were incubated with 0.37 µCi/mL [^3^H]oleic acid and 400 µM oleate complexed with albumin for 4 h. Cellular FFA uptake was measured by determination of [^3^H]oleic acid cellular uptake after extensive cell washing. **B.** TG synthesis. Mature primary WT and CD36 KO adipocytes were incubated with 0.37 µCi/mL [^3^H]oleic acid and 400 µM oleate complexed with albumin in the presence of 0.6 mM DEUP (diethylumbelliferyl phosphate) to inhibit TG hydrolysis. Cellular protein was determined and cellular lipids were extracted and separated by thin-layer chromatography. TG synthesis was determined by measuring the incorporation of [^3^H]oleic acid into TG. Values were normalized to cellular protein. Values shown are mean ± SD of triplicate determinations. Where not visible, error bars are contained within symbols. *, p<0.05, **, p<0.001; WT vs CD36 KO.
(TIF)

Figure S2 **Gene expression in primary adipocytes from WT and CD36 KO mice.** Mature adipocytes were differentiated from the SVF as described in Materials and Methods. RNA was extracted and gene expression was determined by Q-PCR. Data was normalized to 36B4 mRNA. Values shown are mean ± SD (n = 4).
(TIF)

Figure S3 **Daily food intake in WT and CD36 KO mice on a HFD.** WT and CD36 KO mice were placed individually in metabolic cages after 15 wks on a HFD as described in Materials and Methods. Mice were allowed to acclimatize for 1 day. Food intake was recorded for 3 consecutive days. Values shown are mean ± SD (n = 4).
(TIF)

Figure S4 **Improved insulin sensitivity in CD36 KO mice compared to WT mice.** WT and CD36 KO mice were fed a HFD for 15 wks and then fasted for 6 h. Mice were then given a bolus of insulin (1 IU/kg) injection intraperitoneally. Plasma glucose levels were determined at the indicated time points (0, 15, 30, 60 and 90 min). Values shown are mean ± SD (n = 5). Similar results were found in a separate experiment.
(TIF)

Figure S5 **Metabolic parameters in WT and CD36 KO mice on a chow diet.** WT and CD36 KO mice were maintained on a chow diet for 24 wks. **A, B.** Lean and fat body mass were determined by MRI. **C.** Fasting plasma lipids were determined after 6 h of fasting using commercially available kits (Wako). **D.** GTT: Mice were given a bolus of D-glucose (2 g/kg body weight) intraperitoneally after a 6 h fast and blood glucose levels were determined at indicated time points (area under the curve, p<0.05). Values shown are mean ± SD (n = 5).
(TIF)

Figure S6 **Adipose tissue macrophage infiltration and inflammatory gene expression. A.** F4/80 expression in adipose tissue of mice on a chow diet. F4/80 stained epididymal fat sections from WT and CD36 KO mice after 24 wks on a chow diet. F4/80 positive macrophages are shown staining brown. Scale bar, 200 µm (top panel), 100 µm (bottom panel). **B.** Adipose tissue gene expression. RNA was extracted and gene expression was determined by Q-PCR. Data was normalized to 36B4 mRNA. Values shown are mean ± SD (n = 5).
(TIF)

Figure S7 **CD36 promotes pro-inflammatory, pro-apoptotic and ER stress gene expression in mouse peritoneal macrophages.** Peritoneal macrophages were harvested from WT and CD36 KO mice. Macrophages were differentiated in L-cell conditioned medium for 48 h after which cells were treated with LPS (20 ng/mL) for 2 h. Cellular RNA was extracted and gene expression was determined by Q-PCR. Values shown are mean ± SD (n = 4), *, p<0.05; **, p<0.01. Similar results were found in two separate experiments.
(TIF)

Figure S8 **Macrophage and adipocyte specific gene expression in contact co-cultures.** Primary adipocytes were differentiated from the SVF of WT and CD36 KO mice. Peritoneal macrophages isolated from WT and CD36 KO mice were layered and cultured on differentiated adipocytes and co-cultured for 16 h. Cultures were then incubated with LPS (10 ng/mL) for 4 h. Cellular RNA was extracted and gene expression was determined by Q-PCR normalized to 36B4 mRNA. Values shown are mean ± SD (n = 4).
(TIF)

## References

[1] Horng T, Hotamisligil GS (2011). Linking the inflammasome to obesity-related disease.. Nat Med.

[2] Weisberg SP, McCann D, Desai M, Rosenbaum M, Leibel RL (2003). Obesity is associated with macrophage accumulation in adipose tissue.. J Clin Invest.

[3] Xu H, Barnes GT, Yang Q, Tan G, Yang D (2003). Chronic inflammation in fat plays a crucial role in the development of obesity-related insulin resistance.. J Clin Invest.

[4] Hummasti S, Hotamisligil GS (2010). Endoplasmic reticulum stress and inflammation in obesity and diabetes.. Circ Res.

[5] Sun K, Kusminski CM, Scherer PE (2011). Adipose tissue remodeling and obesity.. J Clin Invest.

[6] Cinti S, Mitchell G, Barbatelli G, Murano I, Ceresi E (2005). Adipocyte death defines macrophage localization and function in adipose tissue of obese mice and humans.. J Lipid Res.

[7] Strissel KJ, Stancheva Z, Miyoshi H, Perfield JW, 2nd, DeFuria J (2007). Adipocyte death, adipose tissue remodeling, and obesity complications.. Diabetes.

[8] Murano I, Barbatelli G, Parisani V, Latini C, Muzzonigro G (2008). Dead adipocytes, detected as crown-like structures, are prevalent in visceral fat depots of genetically obese mice.. J Lipid Res.

[9] Takahashi K, Mizuarai S, Araki H, Mashiko S, Ishihara A (2003). Adiposity elevates plasma MCP-1 levels leading to the increased CD11b-positive monocytes in mice.. J Biol Chem.

[10] Fischer-Posovszky P, Wang QA, Asterholm IW, Rutkowski JM, Scherer PE (2011). Targeted Deletion of Adipocytes by Apoptosis Leads to Adipose Tissue Recruitment of Alternatively Activated M2 Macrophages..

[11] Alkhouri N, Gornicka A, Berk MP, Thapaliya S, Dixon LJ (2010). Adipocyte apoptosis, a link between obesity, insulin resistance, and hepatic steatosis.. J Biol Chem.

[12] Silverstein RL, Febbraio M (2009). CD36, a scavenger receptor involved in immunity, metabolism, angiogenesis, and behavior.. Sci Signal.

[13] Endemann G, Stanton LW, Madden KS, Bryant CM, White RT (1993). CD36 is a receptor for oxidized low density lipoprotein.. J Biol Chem.

[14] Febbraio M, Podrez EA, Smith JD, Hajjar DP, Hazen SL (2000). Targeted disruption of the class B scavenger receptor CD36 protects against atherosclerotic lesion development in mice.. J Clin Invest.

[15] Podrez EA, Febbraio M, Sheibani N, Schmitt D, Silverstein RL (2000). Macrophage scavenger receptor CD36 is the major receptor for LDL modified by monocyte-generated reactive nitrogen species.. J Clin Invest.

[16] Janabi M, Yamashita S, Hirano K, Sakai N, Hiraoka H (2000). Oxidized LDL-induced NF-kappa B activation and subsequent expression of proinflammatory genes are defective in monocyte-derived macrophages from CD36-deficient patients.. Arterioscler Thromb Vasc Biol.

[17] Park YM, Febbraio M, Silverstein RL (2009). CD36 modulates migration of mouse and human macrophages in response to oxidized LDL and may contribute to macrophage trapping in the arterial intima.. J Clin Invest.

[18] Lorenz E (2006). TLR2 and TLR4 expression during bacterial infections.. Curr Pharm Des.

[19] Han J, Ulevitch RJ (2005). Limiting inflammatory responses during activation of innate immunity.. Nat Immunol.

[20] Baranova IN, Kurlander R, Bocharov AV, Vishnyakova TG, Chen Z (2008). Role of human CD36 in bacterial recognition, phagocytosis, and pathogen-induced JNK-mediated signaling.. J Immunol.

[21] Greenberg ME, Sun M, Zhang R, Febbraio M, Silverstein R (2006). Oxidized phosphatidylserine-CD36 interactions play an essential role in macrophage-dependent phagocytosis of apoptotic cells.. J Exp Med.

[22] Seimon TA, Nadolski MJ, Liao X, Magallon J, Nguyen M (2010). Atherogenic lipids and lipoproteins trigger CD36-TLR2-dependent apoptosis in macrophages undergoing endoplasmic reticulum stress.. Cell Metab.

[23] Kuang M, Febbraio M, Wagg C, Lopaschuk GD, Dyck JR (2004). Fatty acid translocase/CD36 deficiency does not energetically or functionally compromise hearts before or after ischemia.. Circulation.

[24] Koonen DP, Glatz JF, Bonen A, Luiken JJ (2005). Long-chain fatty acid uptake and FAT/CD36 translocation in heart and skeletal muscle.. Biochim Biophys Acta.

[25] Chen M, Yang Y, Braunstein E, Georgeson KE, Harmon CM (2001). Gut expression and regulation of FAT/CD36: possible role in fatty acid transport in rat enterocytes.. Am J Physiol Endocrinol Metab.

[26] Bonen A, Tandon NN, Glatz JF, Luiken JJ, Heigenhauser GJ (2006). The fatty acid transporter FAT/CD36 is upregulated in subcutaneous and visceral adipose tissues in human obesity and type 2 diabetes.. Int J Obes (Lond).

[27] Coburn CT, Knapp FF, Febbraio M, Beets AL, Silverstein RL (2000). Defective uptake and utilization of long chain fatty acids in muscle and adipose tissues of CD36 knockout mice.. J Biol Chem.

[28] Hajri T, Hall AM, Jensen DR, Pietka TA, Drover VA (2007). CD36-facilitated fatty acid uptake inhibits leptin production and signaling in adipose tissue.. Diabetes.

[29] Nicholls HT, Kowalski G, Kennedy DJ, Risis S, Zaffino LA (2011). Hematopoietic cell-restricted deletion of CD36 reduces high-fat diet-induced macrophage infiltration and improves insulin signaling in adipose tissue.. Diabetes.

[30] Kennedy DJ, Kuchibhotla S, Westfall KM, Silverstein RL, Morton RE (2011). A CD36-dependent pathway enhances macrophage and adipose tissue inflammation and impairs insulin signalling.. Cardiovasc Res.

[31] Crocker PR, Gordon S (1985). Isolation and characterization of resident stromal macrophages and hematopoietic cell clusters from mouse bone marrow.. J Exp Med.

[32] Permana PA, Nair S, Lee YH, Luczy-Bachman G, Vozarova De Courten B (2004). Subcutaneous abdominal preadipocyte differentiation in vitro inversely correlates with central obesity.. Am J Physiol Endocrinol Metab.

[33] Suganami T, Tanimoto-Koyama K, Nishida J, Itoh M, Yuan X (2007). Role of the Toll-like receptor 4/NF-kappaB pathway in saturated fatty acid-induced inflammatory changes in the interaction between adipocytes and macrophages.. Arterioscler Thromb Vasc Biol.

[34] Nishimura S, Manabe I, Nagasaki M, Eto K, Yamashita H (2009). CD8+ effector T cells contribute to macrophage recruitment and adipose tissue inflammation in obesity.. Nat Med.

[35] Feuerer M, Herrero L, Cipolletta D, Naaz A, Wong J (2009). Lean, but not obese, fat is enriched for a unique population of regulatory T cells that affect metabolic parameters.. Nat Med.

[36] Green DR (1998). Apoptotic pathways: the roads to ruin.. Cell.

[37] Guicciardi ME, Gores GJ (2009). Life and death by death receptors.. FASEB J.

[38] Stuart LM, Deng J, Silver JM, Takahashi K, Tseng AA (2005). Response to Staphylococcus aureus requires CD36-mediated phagocytosis triggered by the COOH-terminal cytoplasmic domain.. J Cell Biol.

[39] Furuhashi M, Fucho R, Gorgun CZ, Tuncman G, Cao H (2008). Adipocyte/macrophage fatty acid-binding proteins contribute to metabolic deterioration through actions in both macrophages and adipocytes in mice.. J Clin Invest.

[40] Goudriaan JR, Dahlmans VE, Febbraio M, Teusink B, Romijn JA (2002). Intestinal lipid absorption is not affected in CD36 deficient mice.. Mol Cell Biochem.

[41] Nguyen DV, Drover VA, Knopfel M, Dhanasekaran P, Hauser H (2009). Influence of class B scavenger receptors on cholesterol flux across the brush border membrane and intestinal absorption.. J Lipid Res.

[42] Nauli AM, Nassir F, Zheng S, Yang Q, Lo CM (2006). CD36 is important for chylomicron formation and secretion and may mediate cholesterol uptake in the proximal intestine.. Gastroenterology.

[43] Wueest S, Rapold RA, Schumann DM, Rytka JM, Schildknecht A (2010). Deletion of Fas in adipocytes relieves adipose tissue inflammation and hepatic manifestations of obesity in mice.. J Clin Invest.

[44] Spalding KL, Arner E, Westermark PO, Bernard S, Buchholz BA (2008). Dynamics of fat cell turnover in humans.. Nature.

[45] Hotamisligil GS (2010). Endoplasmic reticulum stress and the inflammatory basis of metabolic disease.. Cell.

[46] Rosen ED, Sarraf P, Troy AE, Bradwin G, Moore K (1999). PPAR gamma is required for the differentiation of adipose tissue in vivo and in vitro.. Mol Cell.

[47] Tontonoz P, Spiegelman BM (2008). Fat and beyond: the diverse biology of PPARgamma.. Annu Rev Biochem.

