# Supplementary figures and images for: Scavenger Receptor CD36 Expression Contributes to Adipose Tissue Inflammation and Cell Death in Diet-Induced Obesity
Source: PLoS One. 2012 May 16;7(5):e36785. doi: 10.1371/journal.pone.0036785 (PMC3353961; doi:10.1371/journal.pone.0036785)

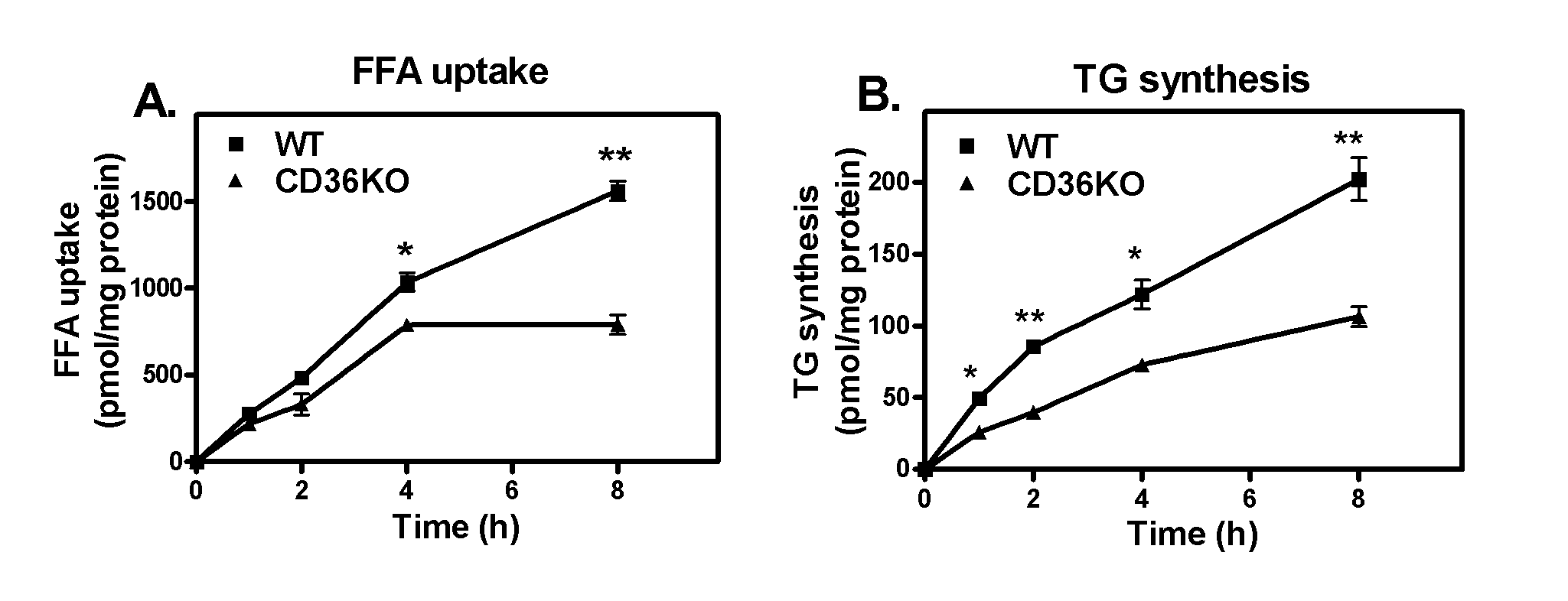

Supplement: Figure S1 — Reduced FFA uptake and triglyceride synthesis in CD36-deficient adipocytes compared to WT control. A. FFA uptake. Mature primary WT and CD36 KO adipocytes were incubated with 0.37 µCi/mL [3H]oleic acid and 400 µM oleate complexed with albumin for 4 h. Cellular FFA uptake was measured by determination of [3H]oleic acid cellular uptake after extensive cell washing. B. TG synthesis. Mature primary WT and CD36 KO adipocytes were incubated with 0.37 µCi/mL [3H]oleic acid and 400 µM oleate complexed with albumin in the presence of 0.6 mM DEUP (diethylumbelliferyl phosphate) to inhibit TG hydrolysis. Cellular protein was determined and cellular lipids were extracted and separated by thin-layer chromatography. TG synthesis was determined by measuring the incorporation of [3H]oleic acid into TG. Values were normalized to cellular protein. Values shown are mean ± SD of triplicate determinations. Where not visible, error bars are contained within symbols. *, p<0.05, **, p<0.001; WT vs CD36 KO. (TIF) [file pone.0036785.s001.tif]

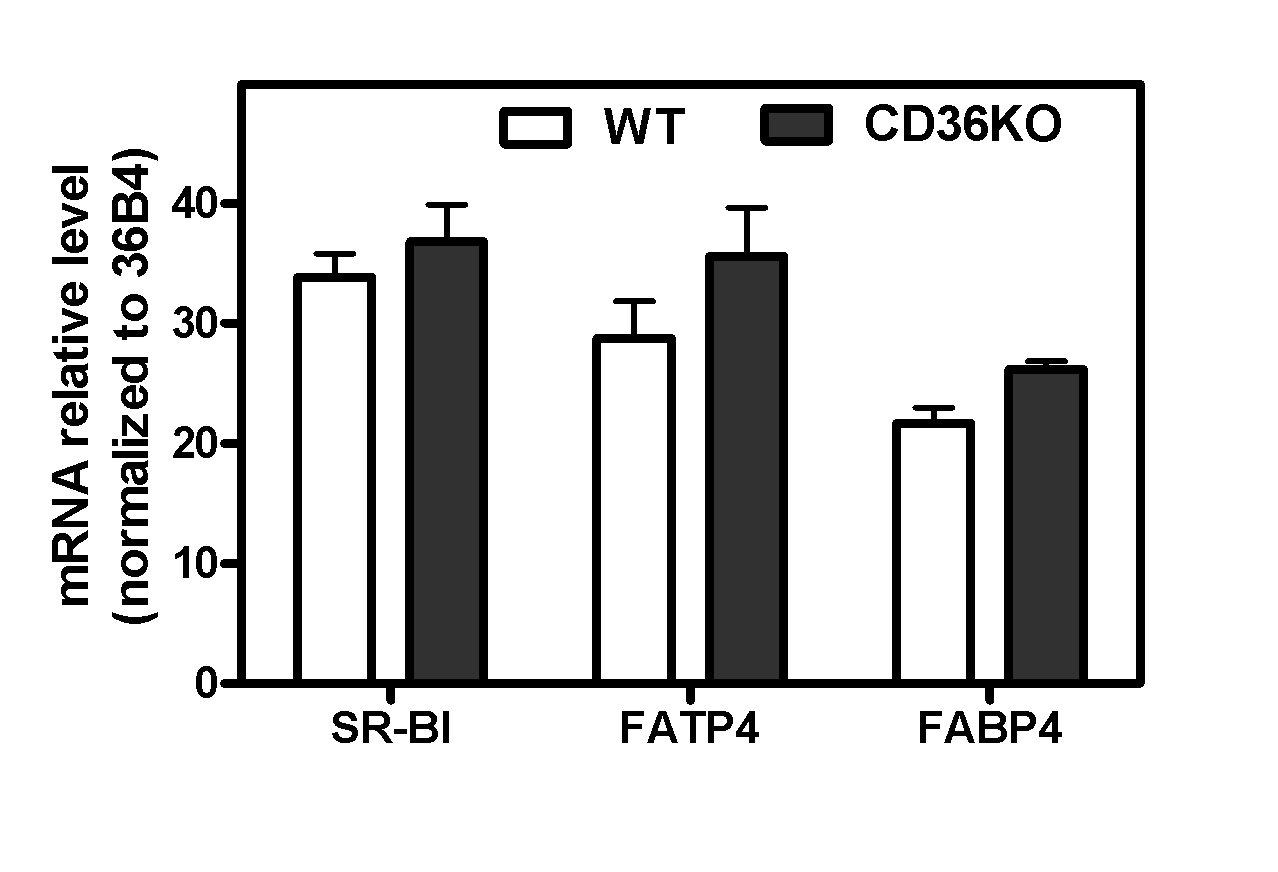

Supplement: Figure S2 — Gene expression in primary adipocytes from WT and CD36 KO mice. Mature adipocytes were differentiated from the SVF as described in Materials and Methods. RNA was extracted and gene expression was determined by Q-PCR. Data was normalized to 36B4 mRNA. Values shown are mean ± SD (n = 4). (TIF) [file pone.0036785.s002.tif]

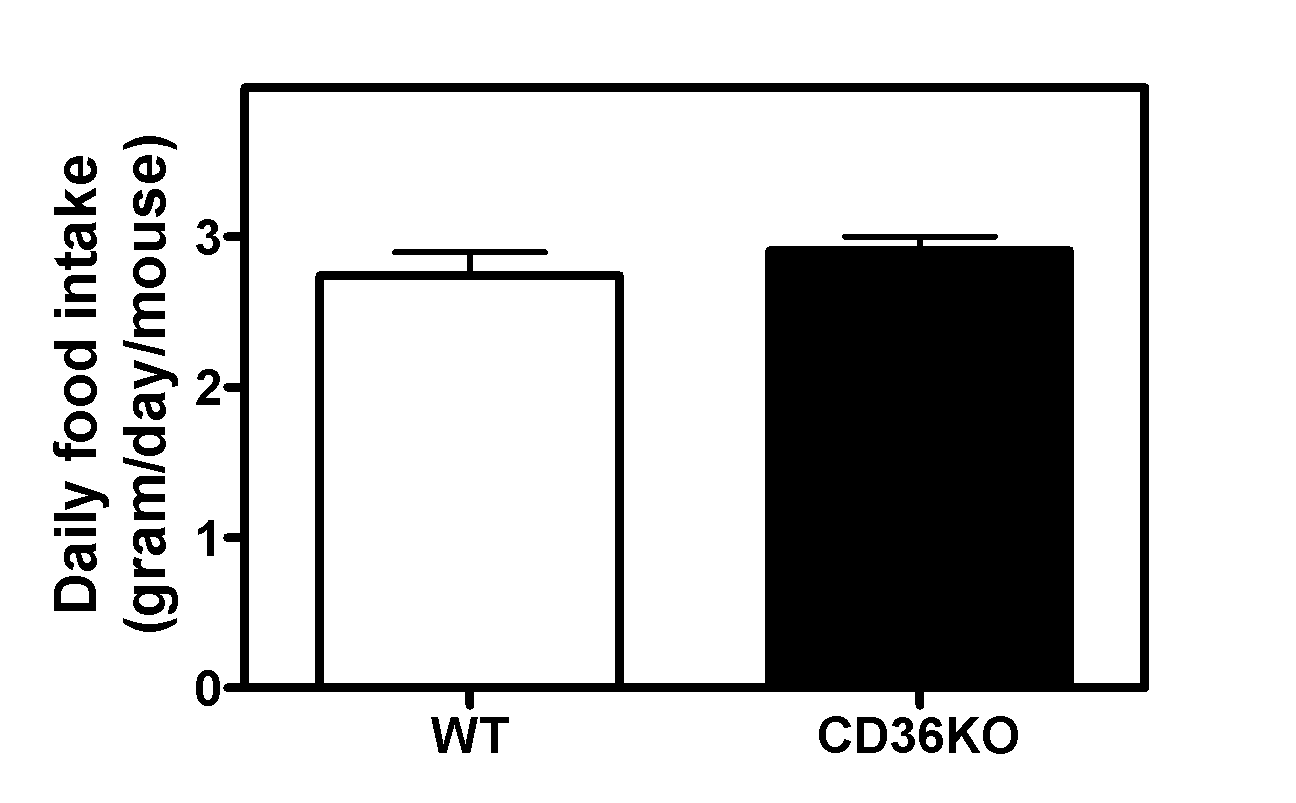

Supplement: Figure S3 — Daily food intake in WT and CD36 KO mice on a HFD. WT and CD36 KO mice were placed individually in metabolic cages after 15 wks on a HFD as described in Materials and Methods. Mice were allowed to acclimatize for 1 day. Food intake was recorded for 3 consecutive days. Values shown are mean ± SD (n = 4). (TIF) [file pone.0036785.s003.tif]

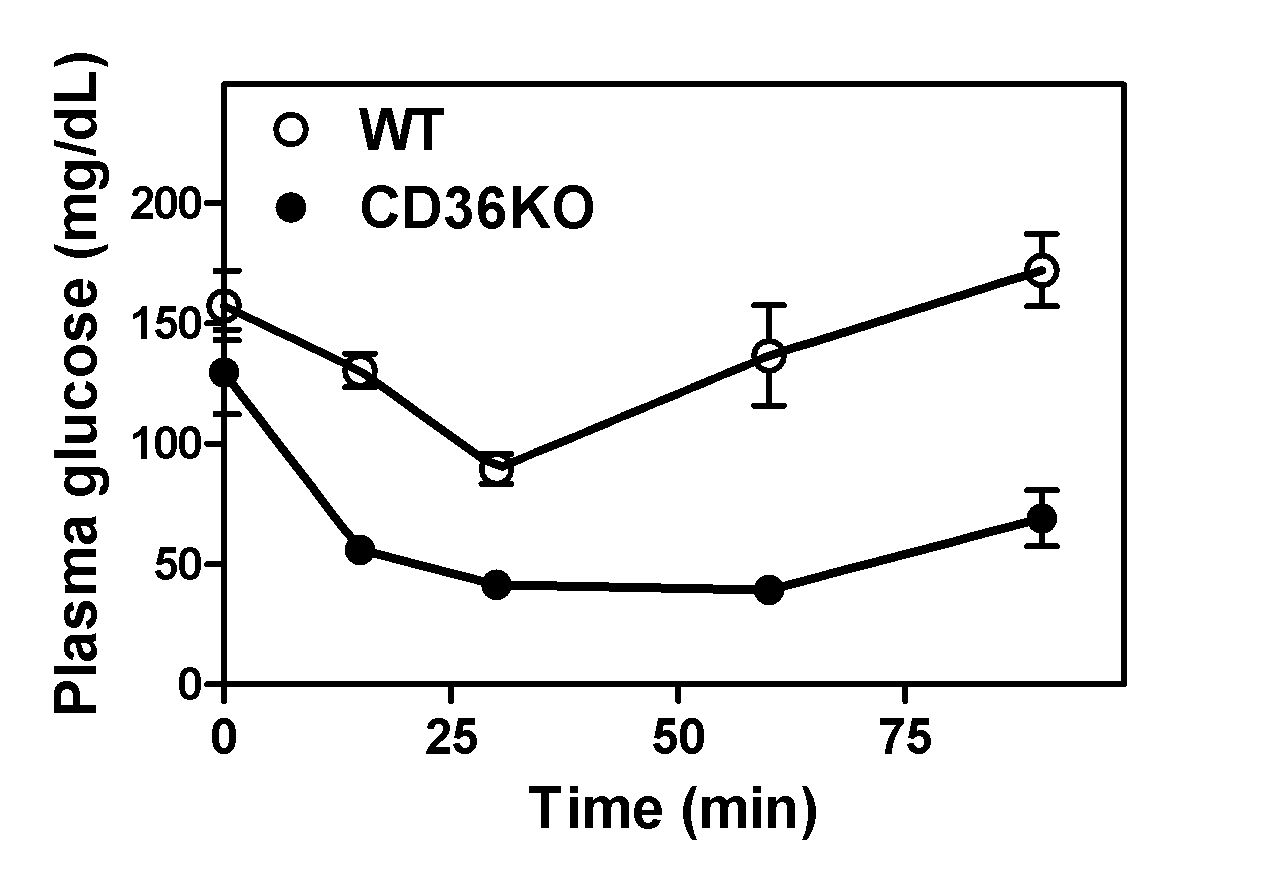

Supplement: Figure S4 — Improved insulin sensitivity in CD36 KO mice compared to WT mice. WT and CD36 KO mice were fed a HFD for 15 wks and then fasted for 6 h. Mice were then given a bolus of insulin (1 IU/kg) injection intraperitoneally. Plasma glucose levels were determined at the indicated time points (0, 15, 30, 60 and 90 min). Values shown are mean ± SD (n = 5). Similar results were found in a separate experiment. (TIF) [file pone.0036785.s004.tif]

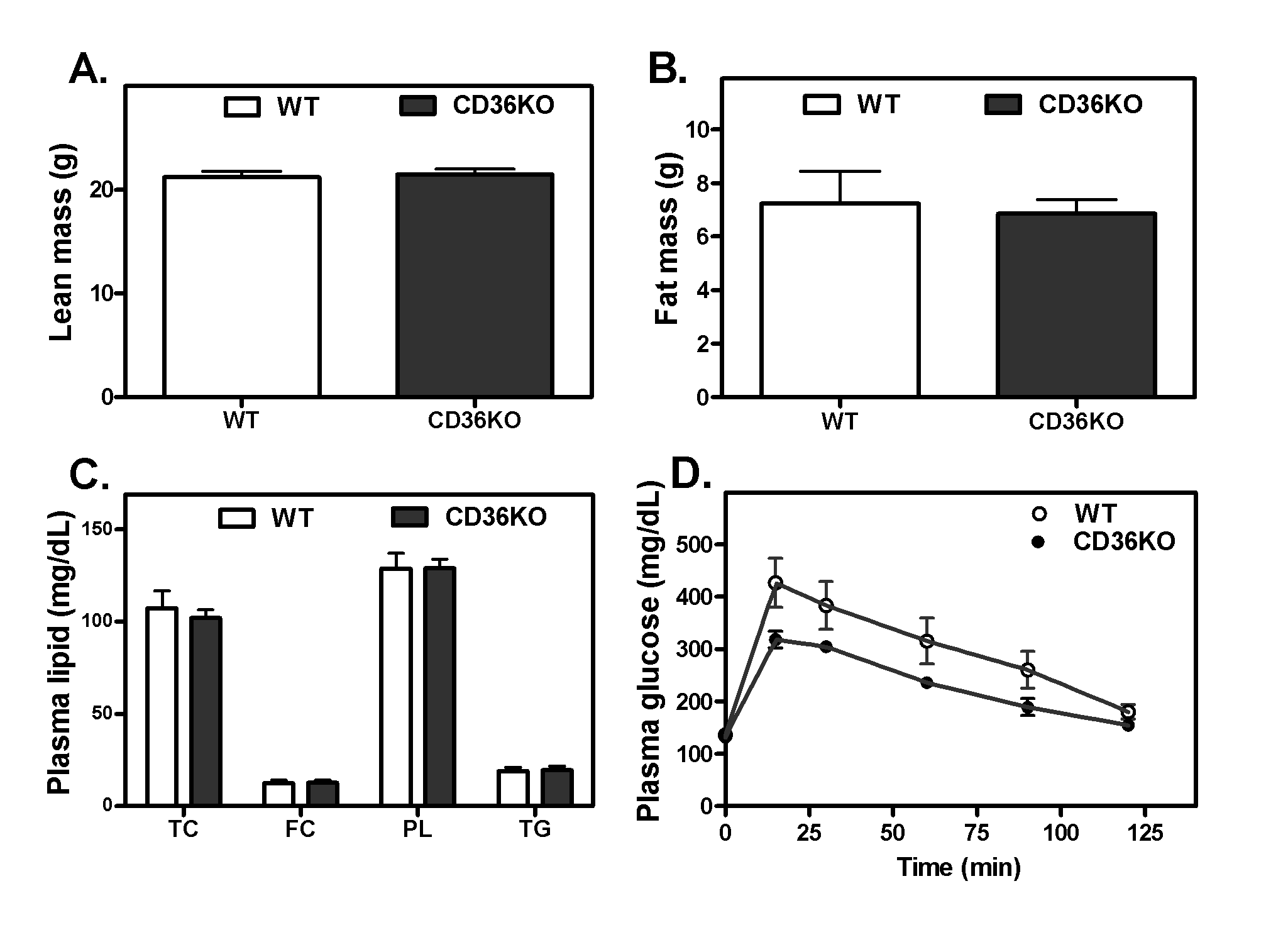

Supplement: Figure S5 — Metabolic parameters in WT and CD36 KO mice on a chow diet. WT and CD36 KO mice were maintained on a chow diet for 24 wks. A, B. Lean and fat body mass were determined by MRI. C. Fasting plasma lipids were determined after 6 h of fasting using commercially available kits (Wako). D. GTT: Mice were given a bolus of D-glucose (2 g/kg body weight) intraperitoneally after a 6 h fast and blood glucose levels were determined at indicated time points (area under the curve, p<0.05). Values shown are mean ± SD (n = 5). (TIF) [file pone.0036785.s005.tif]

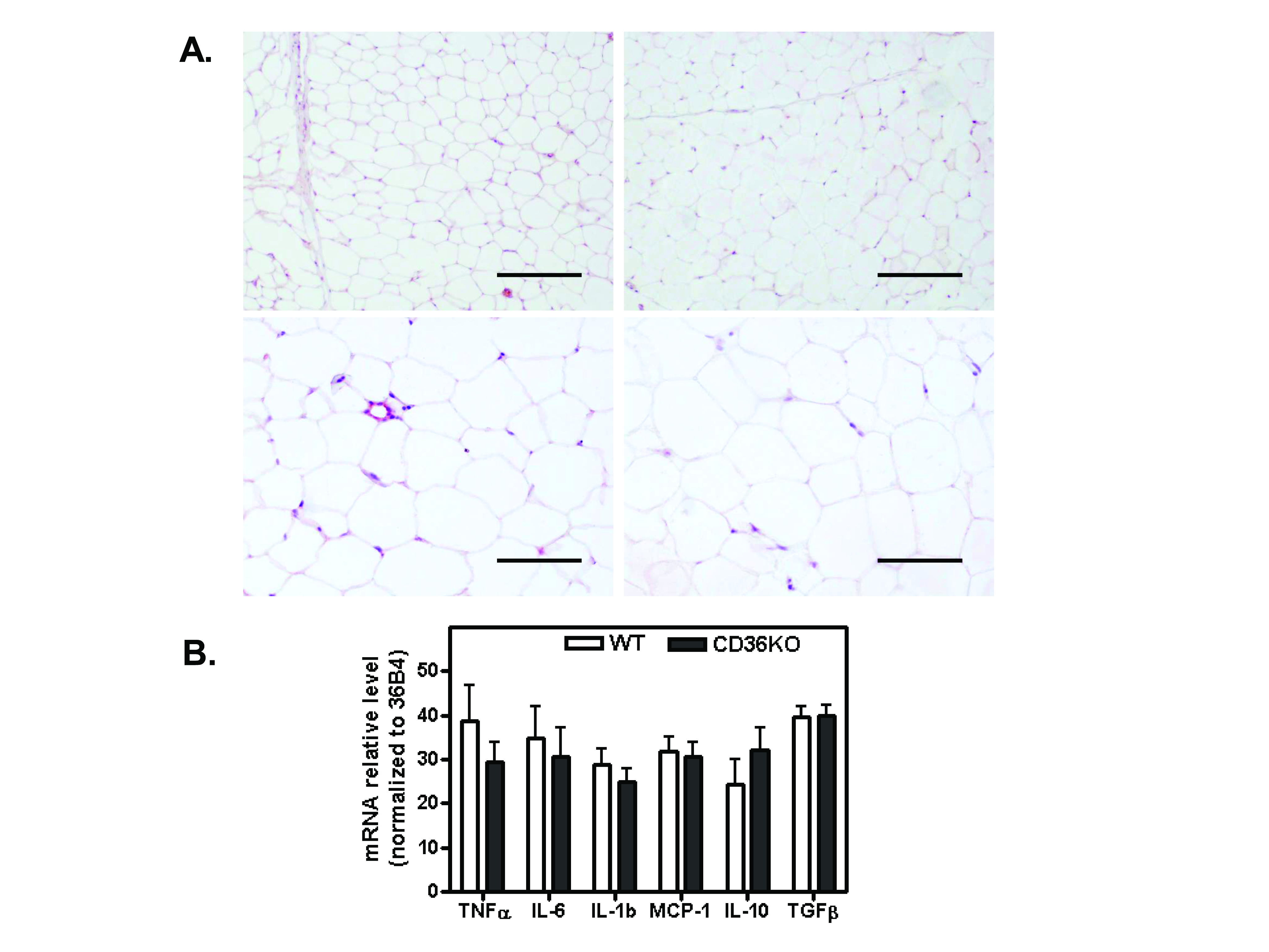

Supplement: Figure S6 — Adipose tissue macrophage infiltration and inflammatory gene expression. A. F4/80 expression in adipose tissue of mice on a chow diet. F4/80 stained epididymal fat sections from WT and CD36 KO mice after 24 wks on a chow diet. F4/80 positive macrophages are shown staining brown. Scale bar, 200 µm (top panel), 100 µm (bottom panel). B. Adipose tissue gene expression. RNA was extracted and gene expression was determined by Q-PCR. Data was normalized to 36B4 mRNA. Values shown are mean ± SD (n = 5). (TIF) [file pone.0036785.s006.tif]

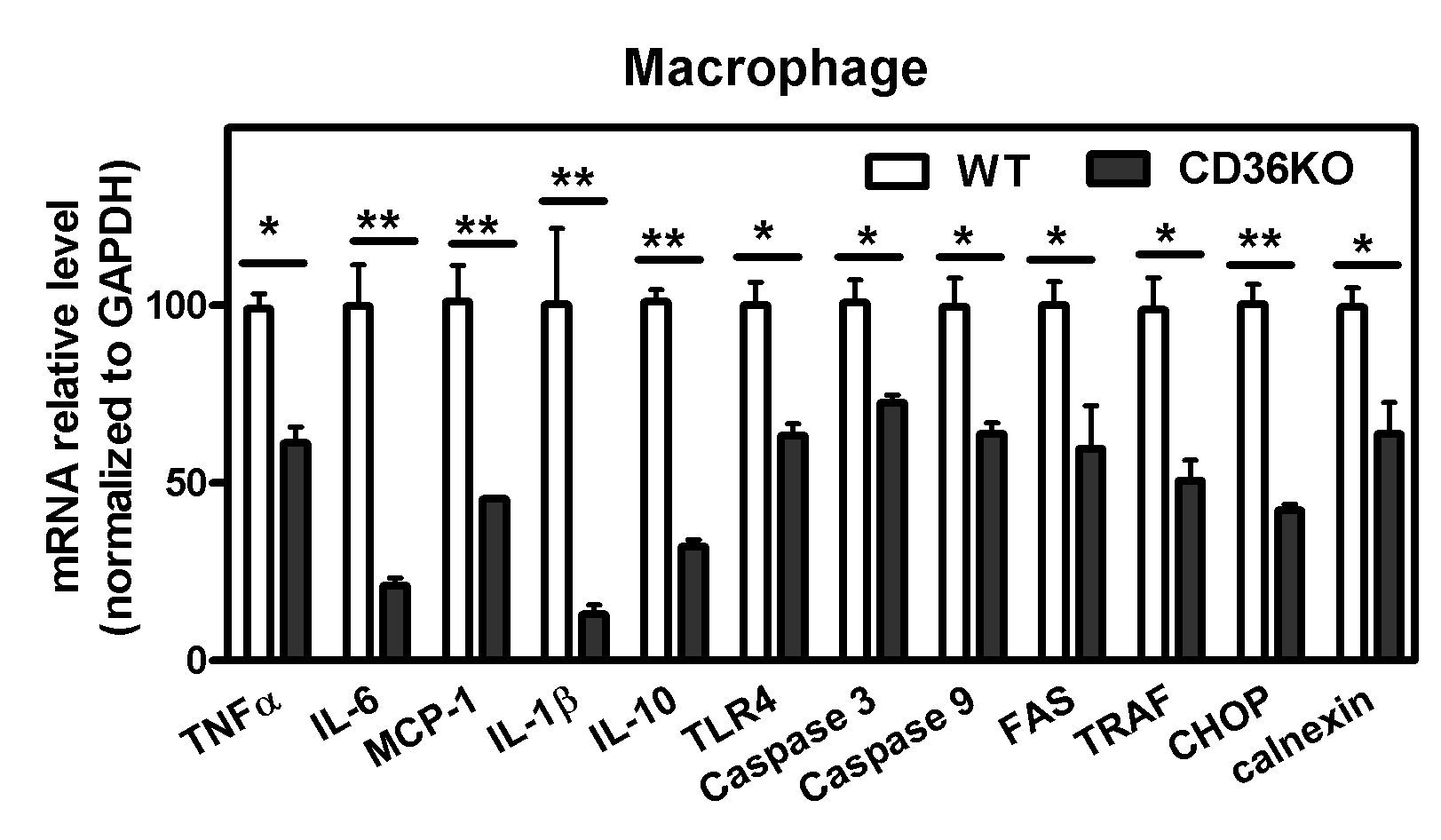

Supplement: Figure S7 — CD36 promotes pro-inflammatory, pro-apoptotic and ER stress gene expression in mouse peritoneal macrophages. Peritoneal macrophages were harvested from WT and CD36 KO mice. Macrophages were differentiated in L-cell conditioned medium for 48 h after which cells were treated with LPS (20 ng/mL) for 2 h. Cellular RNA was extracted and gene expression was determined by Q-PCR. Values shown are mean ± SD (n = 4), *, p<0.05; **, p<0.01. Similar results were found in two separate experiments. (TIF) [file pone.0036785.s007.tif]

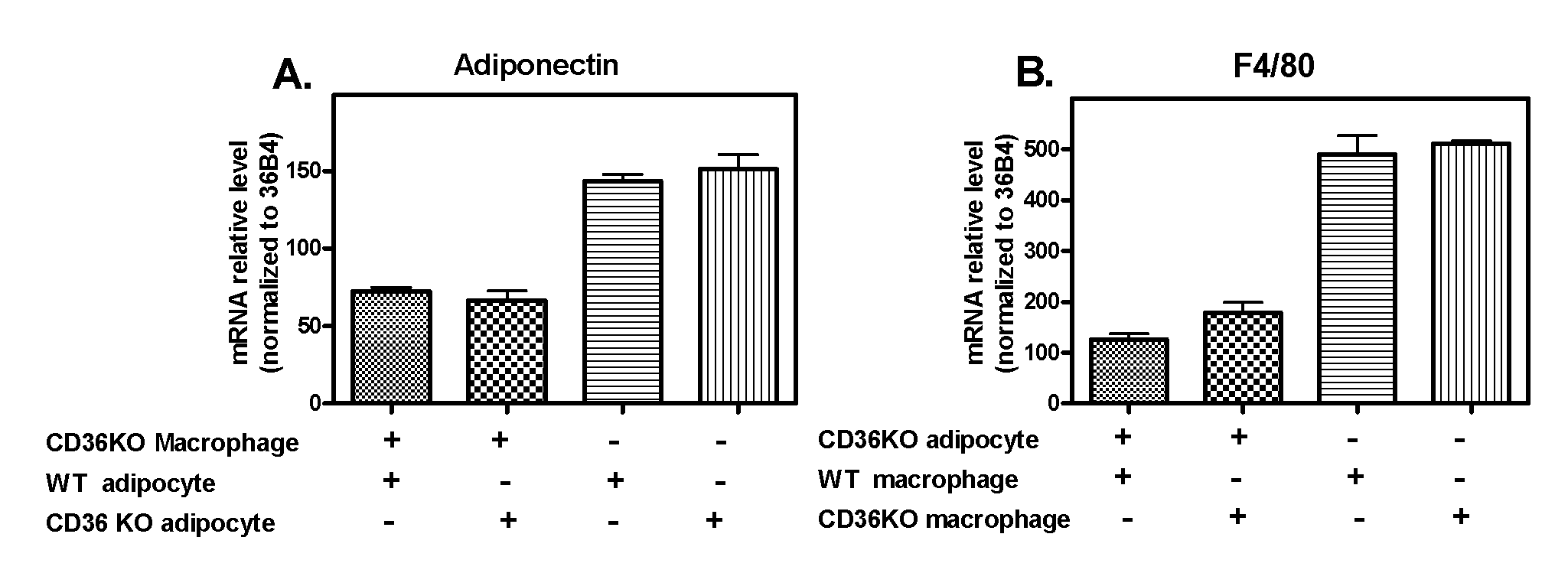

Supplement: Figure S8 — Macrophage and adipocyte specific gene expression in contact co-cultures. Primary adipocytes were differentiated from the SVF of WT and CD36 KO mice. Peritoneal macrophages isolated from WT and CD36 KO mice were layered and cultured on differentiated adipocytes and co-cultured for 16 h. Cultures were then incubated with LPS (10 ng/mL) for 4 h. Cellular RNA was extracted and gene expression was determined by Q-PCR normalized to 36B4 mRNA. Values shown are mean ± SD (n = 4). (TIF) [file pone.0036785.s008.tif]
